# Correction: Tracking the clinico-microbiological profile and molecular characterization of dengue cases during the monsoon-season in Belagavi, Karnataka

**DOI:** 10.1371/journal.pntd.0014049

**Published:** 2026-03-02

**Authors:** Rekha Patil, Asif Kavathekar, Madhav Prabhu, Madhavi Patil, Ameya Kejriwal, Kamran Zaman, Jyothi Bhat, Subarna Roy

There is an error in affiliation 1 for authors Rekha Patil, Madhav Prabhu and Ameya Kejriwal. The correct affiliation 1 is: Department of General Medicine, Jawaharlal Nehru Medical College, KAHER Belagavi, India.
