# Supplementary material for: Tracking the clinico-microbiological profile and molecular characterization of dengue cases during the monsoon-season in Belagavi, Karnataka
Source: PLoS Negl Trop Dis. 2026 Jan 20;20(1):e0013883. doi: 10.1371/journal.pntd.0013883 (PMC12851443; doi:10.1371/journal.pntd.0013883)
Supplement: S1 Table — (DOCX) [file pntd.0013883.s001.docx]

**Supporting information**

**S1 Table: Serotype-specific laboratory data of Complete Blood Count, Liver Function Test, and Renal Function Test & Serotype distribution across thrombocytopenia grades (Tables A and B in S1 Table)**

**Table A: Serotype-specific laboratory data of Complete Blood Count, Liver Function Test, and Renal Function Test**

| **Serotype Vs CBC, LFT & RFT** | **Dengue Serotype 1** | **Dengue Serotype 2** | **Dengue Serotype 3** |
| --- | --- | --- | --- |
| **CBC** | **DEN1 (n=1)** | **DEN2 (n=21) [Mean (SD)]** | **DEN3 (n=1)** |
| **HB (g/dL)** | 16.00 | 13.83 (2.67) | 17.80 |
| **TC (10³/µL)** | 2.20 | 5.13 (3.56) | NA |
| **Platelet Count** | 122000.00 | 114142.86 | 20000.00 |
|  |  |  |  |
| **LFT** | **DEN1 (n=1)** | **DEN2 (n=21) [Median (Q1, Q3)]** | **DEN3 (n=1)** |
| **Total Bilirubin (mg/dl)** | 0.51 | 0.59 (0.44,0.85) | 0.81 |
| **Direct Bilirubin (mg/dl)** | 0.30 | 0.27 (0.23, 0.40) | 0.32 |
| **SGOT (U/L)** | 86.00 | 60.00 (30.00, 137.00) | 97.00 |
| **SGPT (U/L)** | 60.00 | 47.00 (21.00, 107.00) | 112.00 |
| **ALP (U/L)** | 89.00 | 70.00 (60.00, 91.00) | 136.00 |
|  |  |  |  |
| **RFT** | **DEN1 (n=1)** | **DEN2 (n=21) [Mean (SD)]** | **DEN3 (n=1)** |
| **CREAT (mg/dl)** | 1.16 | 0.93 (0.21) | 0.84 |
| **UREA (mg/dl)** | 11.90 | 28.25 (14.37) | 21.80 |
| **BUN** | 5.56 | 13.20 (6.71) | 10.19 |

**Table B: Serotype distribution across thrombocytopenia grades**

| Serotype Vs Thrombocytopenia Grade | Grade 0 (>150,000) | Grade I  (75,000 – 150,000) | Grade II  (50,000 – 75,000) | Grade III  (25,000 – 50,000) | Grade IV (<25,000) |
| --- | --- | --- | --- | --- | --- |
| DEN1 (n=1) | 0 | 1 | 0 | 0 | 0 |
| DEN2 (n=21) | 6 | 9 | 1 | 1 | 4 |
| DEN3 (n=1) | 0 | 0 | 0 | 0 | 1 |
| Total cases | 6 | 10 | 1 | 1 | 5 |
