# Supplementary material for: Tracking the clinico-microbiological profile and molecular characterization of dengue cases during the monsoon-season in Belagavi, Karnataka
Source: PLoS Negl Trop Dis. 2026 Jan 20;20(1):e0013883. doi: 10.1371/journal.pntd.0013883 (PMC12851443; doi:10.1371/journal.pntd.0013883)
Supplement: S2 Table — (DOCX) [file pntd.0013883.s002.docx]

**Supporting information**

**S2 Table:** **Table depicting the estimates of evolutionary divergence between sequences analysed**

|  | 24062_Belagavi_2024 | OQ842500_Bhopal_ 2023 | OR888735_Bhopal_2023 | OM639984_Pune_2022 | OR836573_Shivamogga_2023 | PP968394_Gorakhpur_2024 | PP658562_Varanasi_2024 | OQ15527_Uttrakhand_2023 | PP757871_Raipur_2024 | OP921000_Bengaluru_2022 | PP419020_Warangal_2024 | GU968539_Kerala_2014 | FJ467493_Malaysia_2009 | PP697467_Japan_2024 | OP389112_China_2022 |
| --- | --- | --- | --- | --- | --- | --- | --- | --- | --- | --- | --- | --- | --- | --- | --- |
| 24062_Belagavi_ 2024 |  |  |  |  |  |  |  |  |  |  |  |  |  |  |  |
| OQ842500_Bhopal_2023 | 0.004 |  |  |  |  |  |  |  |  |  |  |  |  |  |  |
| OR888735_Bhopal_2023 | 0.002 | 0.002 |  |  |  |  |  |  |  |  |  |  |  |  |  |
| OM639984_Pune_2022 | 0.002 | 0.002 | 0.000 |  |  |  |  |  |  |  |  |  |  |  |  |
| OR836573_Shivamogga_2023 | 0.004 | 0.000 | 0.002 | 0.002 |  |  |  |  |  |  |  |  |  |  |  |
| PP968394_Gorakhpur_2024 | 0.002 | 0.002 | 0.000 | 0.000 | 0.002 |  |  |  |  |  |  |  |  |  |  |
| PP658562_Varanasi_2024 | 0.002 | 0.002 | 0.000 | 0.000 | 0.002 | 0.000 |  |  |  |  |  |  |  |  |  |
| OQ15527_Uttrakhand_2023 | 0.004 | 0.000 | 0.002 | 0.002 | 0.000 | 0.002 | 0.002 |  |  |  |  |  |  |  |  |
| PP757871_Raipur_2024 | 0.002 | 0.002 | 0.000 | 0.000 | 0.002 | 0.000 | 0.000 | 0.002 |  |  |  |  |  |  |  |
| OP921000_Bengaluru_2022 | 0.002 | 0.002 | 0.000 | 0.000 | 0.002 | 0.000 | 0.000 | 0.002 | 0.000 |  |  |  |  |  |  |
| PP419020_Warangal_2024 | 0.002 | 0.002 | 0.000 | 0.000 | 0.002 | 0.000 | 0.000 | 0.002 | 0.000 | 0.000 |  |  |  |  |  |
| GU968539_Kerala_2014 | 0.090 | 0.085 | 0.087 | 0.087 | 0.085 | 0.087 | 0.087 | 0.085 | 0.087 | 0.087 | 0.087 |  |  |  |  |
| FJ467493_Malaysia_2009 | 0.211 | 0.208 | 0.208 | 0.208 | 0.208 | 0.208 | 0.208 | 0.208 | 0.208 | 0.208 | 0.208 | 0.210 |  |  |  |
| PP697467_Japan_2024 | 0.002 | 0.002 | 0.000 | 0.000 | 0.002 | 0.000 | 0.000 | 0.002 | 0.000 | 0.000 | 0.000 | 0.087 | 0.208 |  |  |
| OP389112_China_2022 | 0.002 | 0.002 | 0.000 | 0.000 | 0.002 | 0.000 | 0.000 | 0.002 | 0.000 | 0.000 | 0.000 | 0.087 | 0.208 | 0.000 |  |
