# Supplementary material for: Tracking the clinico-microbiological profile and molecular characterization of dengue cases during the monsoon-season in Belagavi, Karnataka
Source: PLoS Negl Trop Dis. 2026 Jan 20;20(1):e0013883. doi: 10.1371/journal.pntd.0013883 (PMC12851443; doi:10.1371/journal.pntd.0013883)
Supplement: S3 Table — (DOCX) [file pntd.0013883.s003.docx]

**GenBank Sequences utilized for phylogenetic analysis**

The sequence has been submitted to the GenBank database and is available with accession number PV992631 and the reference sequences utilized for the phylogenetic tree are available in the GenBank database with accession numbers: PP658562, PP757871, PP968394, OM639984, OR888735, OP921000, PP419020, PP697467, OP389112, OQ842500, OR836573, OQ155275, GU968539, FJ467493. '

>PV992631 24062_Belagavi_2024

CCGCGTGTCAACTGTGCAGCAGCTGACAAAGAGATTCTCACTTGGAATGCTACAGGGACGAGGACCACTGAAACTGTTCATGGCCCTGGTGGCATTCCTTCGTTTCCTAACAATCCCGCCAACAGCAGGGATATTAAAAAGATGGGGAACAATCAAAAAATCAAAGGCTATCAATGTCTTGAGAGGGTTCAGGAAAGAGATTGGAAGGATGCTTAACATCTTGAACAGGAGACGCAGAACTGTAGGTATAATTATTATGATGATCCCAACAGTGATGGCGTTCCATTTAACCACACGCAATGGAGAACCACACATGATCGTCAGTAGACAAGAGAAAGGGAAAAGTCTTCTGTTTAAAACAGAGAACGGCGTGAATATGTGCACCCTCATGGCCATGGATCTTGGTGAACTGTGTGAAGACACAATCACTTACAATTGTCCTCTTCTCAGGCAGAATGAACCTGAA

>OQ842500 Bhopal_2023

CCGCGTGTCAACTGTGCAGCAGCTGACAAAGAGATTCTCACTTGGAATGCTACAGGGACGAGGACCACTGAAACTGTTCATGGCCCTGGTGGCATTCCTTCGTTTCCTAACAATCCCGCCAACAGCAGGGATATTAAAAAGATGGGGAACAATCAAAAAATCAAAGGCTATCAATGTCTTGAGAGGGTTCAGGAAAGAGATTGGAAGGATGCTTAACATCTTGAACAGGAGACGCAGAACTGCAGGTATAATTATTATGATGATCCCAACAGTGATGGCGTTCCATTTAACCACACGCAATGGAGAACCACACATGATCGTCAGTAGACAAGAGAAAGGGAAAAGTCTTCTGTTTAAAACAGAGAACGGTGTGAATATGTGCACCCTCATGGCCATGGATCTTGGTGAACTGTGTGAAGACACAATCACTTACAATTGTCCTCTTCTCAGGCAGAATGAACCTGAA

>OR888735 Bhopal_2023

CCGCGTGTCAACTGTGCAGCAGCTGACAAAGAGATTCTCACTTGGAATGCTACAGGGACGAGGACCACTGAAACTGTTCATGGCCCTGGTGGCATTCCTTCGTTTCCTAACAATCCCGCCAACAGCAGGGATATTAAAAAGATGGGGAACAATCAAAAAATCAAAGGCTATCAATGTCTTGAGAGGGTTCAGGAAAGAGATTGGAAGGATGCTTAACATCTTGAACAGGAGACGCAGAACTGCAGGTATAATTATTATGATGATCCCAACAGTGATGGCGTTCCATTTAACCACACGCAATGGAGAACCACACATGATCGTCAGTAGACAAGAGAAAGGGAAAAGTCTTCTGTTTAAAACAGAGAACGGCGTGAATATGTGCACCCTCATGGCCATGGATCTTGGTGAACTGTGTGAAGACACAATCACTTACAATTGTCCTCTTCTCAGGCAGAATGAACCAGAA

>OM639984 Pune_2022

CCGCGTGTCAACTGTGCAGCAGCTGACAAAGAGATTCTCACTTGGAATGCTACAGGGACGAGGACCACTGAAACTGTTCATGGCCCTGGTGGCATTCCTTCGTTTCCTAACAATCCCGCCAACAGCAGGGATATTAAAAAGATGGGGAACAATCAAAAAATCAAAGGCTATCAATGTCTTGAGAGGGTTCAGGAAAGAGATTGGAAGGATGCTTAACATCTTGAACAGGAGACGCAGAACTGCAGGTATAATTATTATGATGATCCCAACAGTGATGGCGTTCCATTTAACCACACGCAATGGAGAACCACACATGATCGTCAGTAGACAAGAGAAAGGGAAAAGTCTTCTGTTTAAAACAGAGAACGGCGTGAATATGTGCACCCTCATGGCCATGGATCTTGGTGAACTGTGTGAAGACACAATCACTTACAATTGTCCTCTTCTCAGGCAGAATGAACCAGAA

>OR836573 Shivamogga_2023

CCGCGTGTCAACTGTGCAGCAGCTGACAAAGAGATTCTCACTTGGAATGCTACAGGGACGAGGACCACTGAAACTGTTCATGGCCCTGGTGGCATTCCTTCGTTTCCTAACAATCCCGCCAACAGCAGGGATATTAAAAAGATGGGGAACAATCAAAAAATCAAAGGCTATCAATGTCTTGAGAGGGTTCAGGAAAGAGATTGGAAGGATGCTTAACATCTTGAACAGGAGACGCAGAACTGCAGGTATAATTATTATGATGATCCCAACAGTGATGGCGTTCCATTTAACCACACGCAATGGAGAACCACACATGATCGTCAGTAGACAAGAGAAAGGGAAAAGTCTTCTGTTTAAAACAGAGAACGGTGTGAATATGTGCACCCTCATGGCCATGGATCTTGGTGAACTGTGTGAAGACACAATCACTTACAATTGTCCTCTTCTCAGGCAGAATGAACCTGAA

>PP968394 Gorakhpur_2024

CCGCGTGTCAACTGTGCAGCAGCTGACAAAGAGATTCTCACTTGGAATGCTACAGGGACGAGGACCACTGAAACTGTTCATGGCCCTGGTGGCATTCCTTCGTTTCCTAACAATCCCGCCAACAGCAGGGATATTAAAAAGATGGGGAACAATCAAAAAATCAAAGGCTATCAATGTCTTGAGAGGGTTCAGGAAAGAGATTGGAAGGATGCTTAACATCTTGAACAGGAGACGCAGAACTGCAGGTATAATTATTATGATGATCCCAACAGTGATGGCGTTCCATTTAACCACACGCAATGGAGAACCACACATGATCGTCAGTAGACAAGAGAAAGGGAAAAGTCTTCTGTTTAAAACAGAGAACGGCGTGAATATGTGCACCCTCATGGCCATGGATCTTGGTGAACTGTGTGAAGACACAATCACTTACAATTGTCCTCTTCTCAGGCAGAATGAACCTGAA

>PP658562 Varanasi_2024

CCGCGTGTCAACTGTGCAGCAGCTGACAAAGAGATTCTCACTTGGAATGCTACAGGGACGAGGACCACTGAAACTGTTCATGGCCCTGGTGGCATTCCTTCGTTTCCTAACAATCCCGCCAACAGCAGGGATATTAAAAAGATGGGGAACAATCAAAAAATCAAAGGCTATCAATGTCTTGAGAGGGTTCAGGAAAGAGATTGGAAGGATGCTTAACATCTTGAACAGGAGACGCAGAACTGCAGGTATAATTATTATGATGATCCCAACAGTGATGGCGTTCCATTTAACCACACGCAATGGAGAACCACACATGATCGTCAGTAGACAAGAGAAAGGGAAAAGTCTTCTGTTTAAAACAGAGAACGGCGTGAATATGTGCACCCTCATGGCCATGGATCTTGGTGAACTGTGTGAAGACACAATCACTTACAATTGTCCTCTTCTCAGGCAGAATGAACCTGAA

>OQ155275 UTTARAKHAND_2023

CCGCGTGTCAACTGTGCAGCAGCTGACAAAGAGATTCTCACTTGGAATGCTACAGGGACGAGGACCACTGAAACTGTTCATGGCCCTGGTGGCATTCCTTCGTTTCCTAACAATCCCGCCAACAGCAGGGATATTAAAAAGATGGGGAACAATCAAAAAATCAAAGGCTATCAATGTCTTGAGAGGGTTCAGGAAAGAGATTGGAAGGATGCTTAACATCTTGAACAGGAGACGCAGAACTGCAGGTATAATTATTATGATGATCCCAACAGTGATGGCGTTCCATTTAACCACACGCAATGGAGAACCACACATGATCGTCAGTAGACAAGAGAAAGGGAAAAGTCTTCTGTTTAAAACAGAGAACGGTGTGAATATGTGCACCCTCATGGCCATGGATCTTGGTGAACTGTGTGAAGACACAATCACTTACAATTGTCCTCTTCTCAGGCAGAATGAACCTGAA

>PP757871 Raipur_2024

CCGCGTGTCAACTGTGCAGCAGCTGACAAAGAGATTCTCACTTGGAATGCTACAGGGACGAGGACCACTGAAACTGTTCATGGCCCTGGTGGCATTCCTTCGTTTCCTAACAATCCCGCCAACAGCAGGGATATTAAAAAGATGGGGAACAATCAAAAAATCAAAGGCTATCAATGTCTTGAGAGGGTTCAGGAAAGAGATTGGAAGGATGCTTAACATCTTGAACAGGAGACGCAGAACTGCAGGTATAATTATTATGATGATCCCAACAGTGATGGCGTTCCATTTAACCACACGCAATGGAGAACCACACATGATCGTCAGTAGACAAGAGAAAGGGAAAAGTCTTCTGTTTAAAACAGAGAACGGCGTGAATATGTGCACCCTCATGGCCATGGATCTTGGTGAACTGTGTGAAGACACAATCACTTACAATTGTCCTCTTCTCAGGCAGAATGAACCAGAA

>OP921000 Bengaluru_2022

CCGCGTGTCAACTGTGCAGCAGCTGACAAAGAGATTCTCACTTGGAATGCTACAGGGACGAGGACCACTGAAACTGTTCATGGCCCTGGTGGCATTCCTTCGTTTCCTAACAATCCCGCCAACAGCAGGGATATTAAAAAGATGGGGAACAATCAAAAAATCAAAGGCTATCAATGTCTTGAGAGGGTTCAGGAAAGAGATTGGAAGGATGCTTAACATCTTGAACAGGAGACGCAGAACTGCAGGTATAATTATTATGATGATCCCAACAGTGATGGCGTTCCATTTAACCACACGCAATGGAGAACCACACATGATCGTCAGTAGACAAGAGAAAGGGAAAAGTCTTCTGTTTAAAACAGAGAACGGCGTGAATATGTGCACCCTCATGGCCATGGATCTTGGTGAACTGTGTGAAGACACAATCACTTACAATTGTCCTCTTCTCAGGCAGAATGAACCAGAA

>PP419020 WARANGAL_2024

CCGCGTGTCAACTGTGCAGCAGCTGACAAAGAGATTCTCACTTGGAATGCTACAGGGACGAGGACCACTGAAACTGTTCATGGCCCTGGTGGCATTCCTTCGTTTCCTAACAATCCCGCCAACAGCAGGGATATTAAAAAGATGGGGAACAATCAAAAAATCAAAGGCTATCAATGTCTTGAGAGGGTTCAGGAAAGAGATTGGAAGGATGCTTAACATCTTGAACAGGAGACGCAGAACTGCAGGTATAATTATTATGATGATCCCAACAGTGATGGCGTTCCATTTAACCACACGCAATGGAGAACCACACATGATCGTCAGTAGACAAGAGAAAGGGAAAAGTCTTCTGTTTAAAACAGAGAACGGCGTGAATATGTGCACCCTCATGGCCATGGATCTTGGTGAACTGTGTGAAGACACAATCACTTACAATTGTCCTCTTCTCAGGCAGAATGAACC

>GU968539 Kerala_2014

AGAAACCGCGTGTCAACTGTGCAACAGCTGACAAAGAGATTCTCACTTGGAATGCTGCAGGGACGAGGACCGTTGAAACTGTTCATGGCCTTGGTGGCATTCCTTCGTTTCCTAACAATCCCACCAACAGCAGGGATACTAAAAAGATGGGGAACGATCAAAAAGTCAAAAGCCATCAATGTCTTGAGAGGGTTCAGGAAAGAGATTGGAAGGATGTTGAACATCTTGAATAGGAGACGCAGAACTGCAGGCGTGATCATCATGCTAATTCCAACAGCGATGGCGTTCCATTTAACCACACGCAACGGAGAACCACACATGATCGTCAGCAGACAAGAGAAAGGGAAAAGTCTCTTGTTCAAAACAGAGGATGGTGTGAACATGTGTACCCTCATGGCCATGGACCTTGGTGAACTGTGTGAAGACACAATCACTTATAACTGTCCTCTTCTCAGGCAGAATGAACCTGA

>FJ467493 Malaysia 2009

CCGCGTGTCAACGATTCAACAGTTGACGAAGAGATTCTCACTTGGAATGCTACAAGGGAAAGGGCCATTAAAGTTGTTTATGGCCCTAGTGGCCTTCCTTCGTTTCCTAACCATCCCACCAACAGCAGGGATATTGAAGAGATGGGGAACGATTAAAAAATCAAAAGCCATCAATGTGTTAAGAGGATTCAGAAAGGAAATCGGGAGAATGCTGAACATATTGAACAGAAGACGCAGAACAACAGGTCTTCTCATCATGATTATCCCAACAGTGATGGCATTCCACTTGACCACACGCAATGGGGAGCCACACATGATTGTCAGCAAGCACGAGAAAGGAAAGAGCCTCCTCTTCAAAACGGAAGATGGAATGAACATGTGCACCCTCATGGCCATGGATCTGGGCGAGCTGTGTGAGGACACCATTACATACAAGTGTCCTTTCCTCAAGCAGAACGAACCAGAA

>PP697467 Japan_2024

CCGCGTGTCAACTGTGCAGCAGCTGACAAAGAGATTCTCACTTGGAATGCTACAGGGACGAGGACCACTGAAACTGTTCATGGCCCTGGTGGCATTCCTTCGTTTCCTAACAATCCCGCCAACAGCAGGGATATTAAAAAGATGGGGAACAATCAAAAAATCAAAGGCTATCAATGTCTTGAGAGGGTTCAGGAAAGAGATTGGAAGGATGCTTAACATCTTGAACAGGAGACGCAGAACTGCAGGTATAATTATTATGATGATCCCAACAGTGATGGCGTTCCATTTAACCACACGCAATGGAGAACCACACATGATCGTCAGTAGACAAGAGAAAGGGAAAAGTCTTCTGTTTAAAACAGAGAACGGCGTGAATATGTGCACCCTCATGGCCATGGATCTTGGTGAACTGTGTGAAGACACAATCACTTACAATTGTCCTCTTCTCAGGCAGAATGAACCTGAA

>OP389112 China_2022

CCGCGTGTCAACTGTGCAGCAGCTGACAAAGAGATTCTCACTTGGAATGCTACAGGGACGAGGACCACTGAAACTGTTCATGGCCCTGGTGGCATTCCTTCGTTTCCTAACAATCCCGCCAACAGCAGGGATATTAAAAAGATGGGGAACAATCAAAAAATCAAAGGCTATCAATGTCTTGAGAGGGTTCAGGAAAGAGATTGGAAGGATGCTTAACATCTTGAACAGGAGACGCAGAACTGCAGGTATAATTATTATGATGATCCCAACAGTGATGGCGTTCCATTTAACCACACGCAATGGAGAACCACACATGATCGTCAGTAGACAAGAGAAAGGGAAAAGTCTTCTGTTTAAAACAGAGAACGGCGTGAATATGTGCACCCTCATGGCCATGGATCTTGGTGAACTGTGTGAAGACACAATCACTTACAATTGTCCTCTTCTCAGGCAGAATGAACCAGAA
