# Supplementary material for: Tracking the clinico-microbiological profile and molecular characterization of dengue cases during the monsoon-season in Belagavi, Karnataka
Source: PLoS Negl Trop Dis. 2026 Jan 20;20(1):e0013883. doi: 10.1371/journal.pntd.0013883 (PMC12851443; doi:10.1371/journal.pntd.0013883)
Supplement: Attachment — Submitted filename: Review 2.docx [file pntd.0013883.s005.docx]

**Review Comments**

This manuscript entitled “Tackling the clinic-microbiological profile and molecular evaluation of Dengue cases during Monsoon-season in Balagavi, Karnataka” presents a prospective, hospital-based observational study conducted in Belagavi, Karnataka, during the 2024 monsoon season. The authors examined 89 confirmed dengue patients out of 794 dengue-positive cases. Serotyping was performed on NS1-positive samples, revealing DENV-2 as the predominant serotype (91.3%), followed by DENV-1 and DENV-3. Phylogenetic analysis of DENV-2 strains indicated close relationships with isolates from Bhopal, suggesting possible regional transmission dynamics. Clinically, fever and myalgia were predominant, while DENV-2 cases more often presented with chills, headache, and retro-orbital pain. The study highlights late-phase diagnosis (high IgM positivity), raising concerns about delays in detection, and emphasizes the importance of continued molecular surveillance in guiding outbreak preparedness and vaccine strategies.

This study provides important molecular data from northern Karnataka, a region with limited previous reports. It combines clinical, serological, and molecular findings, shows that DENV-2 is the dominant serotype, and highlights implications for diagnosis, surveillance, and vaccine planning.

Comments:-

1. Why were only 28 NS1-positive cases tested by RT-PCR? Could testing additional RNA-extractable samples have given a more complete picture of circulating serotypes?

2. Out of 794 dengue-positive cases, only 28 NS1-positive samples were tested by RT-PCR. Are these few samples really representative of all the cases? Would testing more NS1-positive (or possibly IgM-positive) samples have given stronger and more reliable conclusions about the circulating serotypes?

3. Sequencing Strategy:- Only a subset of RT-PCR positive samples underwent sequencing. Please provide details on how samples were chosen for sequencing and whether this may have biased phylogenetic interpretation.

4. Can the findings from this single tertiary care centre be generalized to the wider population of northern Karnataka?”

4. Clinical Correlations:- The manuscript reports symptom associations with serotypes (e.g., chills, retro-orbital pain with DENV-2). Given the small number of non–DENV-2 cases, how reliable are these associations?

5. What specific factors determined which NS1-positive samples were chosen for further analysis?

6. NS1 antigen is typically detectable only during the first 5 days of illness. After this period, NS1 results may turn negative even in confirmed dengue cases. Do the authors have data on the number of days between symptom onset and sample collection? If available, please include this information to support the explanation for low NS1 positivity

7. Hematological and Biochemical Parameters:- Thrombocytopenia and creatinine levels are mentioned briefly but not fully discussed. Could the authors elaborate on the relevance of hematological and biochemical parameters, and indicate whether any kidney involvement was observed in severe cases?

8. For the samples that were tested for dengue serotypes, can the authors provide the corresponding CBC, RFT, and LFT results? Presenting this data in a table in the supplementary material would help readers see any clinical differences between serotypes.

This is a well-designed and timely study that provides valuable conceptions into dengue surveillance in Karnataka. The manuscript will benefit from clarifications, with these revisions; the paper will be significantly strengthened. I therefore recommend acceptance after minor revisions.
