# Supplementary material for: Tracking the clinico-microbiological profile and molecular characterization of dengue cases during the monsoon-season in Belagavi, Karnataka
Source: PLoS Negl Trop Dis. 2026 Jan 20;20(1):e0013883. doi: 10.1371/journal.pntd.0013883 (PMC12851443; doi:10.1371/journal.pntd.0013883)
Supplement: Attachment — Submitted filename: Response to reviwer & editor_12.11.2025.docx [file pntd.0013883.s007.docx]

**Dear Academic Editor and Reviewers,**

Thank you for your thorough review of our manuscript, **PNTD-D-25-01558, titled "Tracking the Clinico-microbiological profile and molecular evolution of Dengue cases during the Monsoon-Season in Belagavi, Karnataka."** We appreciate the time and expertise you dedicated to evaluating our work.

We are pleased that you recognize the merit of our study, and we have addressed all the scientific, methodological, and administrative concerns raised in the decision letter and by the reviewers. We believe that these revisions have significantly improved the clarity, scientific rigor, and presentation of our findings, enhancing its suitability for publication in PLOS Neglected Tropical Diseases.

Below is our point-by-point response to each comment. All changes have been highlighted in the revised manuscript using Track Changes.

**Response to Editor’s Comments**

1. The paper does not have a clear hypothesis and the results do not support the data presented.

**Response:** We acknowledge that the study lacks a formal, hypothesis-driven design. We have clarified the study's primary objectives as an essential descriptive molecular and clinico-epidemiological surveillance effort for an area (North Karnataka) where such data is critically absent.

The revised Introduction and Abstract now clearly state our two main aims:

1. To identify and characterize the circulating DENV serotypes during the 2024 monsoon season in the study region.
2. To analyze the clinical and demographic profiles of the hospitalized dengue patient cohort and correlate these patterns with the identified serotypes.

We have carefully reviewed the Results and Discussion sections to ensure that conclusions are now directly supported by the data presented, particularly regarding the predominance of DENV-2 and the clinical profile of the large (N=89) hospitalized cohort.

2. The article needs careful copyediting and proof-reading. For example, Table 1 states Igm, when it should be IgM.

**Response:** We sincerely apologize for the typographical errors. The entire manuscript has undergone rigorous copyediting and proofreading. "Igm" has been corrected to "IgM" and all similar errors have been rectified throughout the text, tables, and figures.

3. Please clarify how 89 patients were chosen from the 794 patients with dengue for further analysis. Why were only 28 samples testing for serotyping?

**Response:** We have revised the *Materials and Methods* (Section 2.1) to clarify the selection process: A total of 794 patients were dengue-positive (NS1 and/or IgM) cases identified by routine hospital diagnostics (SD Bioline RDT) during the study period. The 89 cases included in our study represent a consecutive subset of these 794 patients who were admitted to the tertiary care centre (hospitalized cohort) and whose residual samples were subsequently forwarded to the ICMR-NITM VRDL lab for advanced molecular and serological confirmation/serotyping. The study design inherently focuses on the more severe cases requiring hospitalization, providing a targeted snapshot of the circulating DENV in the context of clinical management.

We clarify that a greater number was initially intended. However, serotyping by RT-PCR was performed only on the 28 samples that were NS1 antigen positive. This decision was based on two factors: 1) The NS1-positive samples represent the acute viraemic phase, which offers the highest probability of successful DENV RNA amplification and serotype determination. 2) Limited residual sample volume for molecular analysis after initial hospital diagnostics.

We have revised *Methods* (Section 2.4) to clearly state this rationale and acknowledge it as a limitation.

4. Were there any considerations about false-positivity due to lower specificity of the IgM?

**Response:** We acknowledge that IgM can present cross-reactivity (lower specificity) in secondary infections. However, in our setting, the high IgM positivity and low NS1 positivity suggested that the vast majority of our hospitalized patients presented after day 7 of illness (late viraemic/early recovery phase). In this phase, IgM is the most critical and sensitive diagnostic marker. The initial rapid test screening was confirmed using Panbio Dengue early ELISA and the Dengue IgM capture ELISA kit (ICMR-NIV, Pune), strengthening the diagnostic confidence. We have added a statement in the *Discussion* (Paragraph 2) to reflect this diagnostic strategy, acknowledging the inherent limitation of late presentation to the tertiary centre.

5. I would not include interpretation of results in the results - please use those in the discussion. For example, "suggesting higher vulnerability among young adults" is subjective and debatable. Young adults maybe at higher risk because they are of working age and hence might have a higher exposure.

**Response:** We fully agree with this comment. We have reviewed the entire Results section (Section 3.1) and removed all interpretive language, including the phrase "suggesting higher vulnerability among young adults". All interpretations and speculative statements have been relocated to the Discussion section, where they are appropriately contextualized and discussed within the limitations of the study.

6. If the SD Bio line Dengue Duo assay tested IgG and IgM, why is IgG not reported? For section 3.2, it is unclear which results are from rapid testing and which are from ELISA - I would make that clearer.

**Response:** Initial screening was performed using the SD Bio line Rapid Test at the hospital and our subsequent VRDL analysis focused on NS1 and IgM ELISA (Panbio Dengue early ELISA and Dengue IgM capture ELISA kit) as the gold standard markers for acute/recent infection in clinical management. IgG results were not consistently validated by an accompanying IgG capture ELISA in the VRDL lab and were therefore excluded to ensure data quality and focus on the primary study objective. We have included a sentence in the *Limitations* section to acknowledge the lack of IgG data for classification of primary vs. secondary infections. The data presented in Section 3.2 (Dengue Diagnostic Marker) are the final, confirmed results based on the ELISA testing carried out at the VRDL laboratory. We have made this explicit in the text.

7. IF Figure 1 is supposed to be a bubble plot, please include bubbles and not crosses

**Response:** We apologize for the ambiguous figure style. Figure 1 has been revised to use clear circles (bubbles) instead of the 'X' markers to improve visualization and fully comply with the intention of a bubble plot. The bubble plot was created using the Python package matplotlib v3.9.2

8. Unclear what the relevance of section 3.3 and 3.4. the sample size is not large enough to make conclusions that associate clinical features with a specific serotype.

**Response:** Sections 3.3 (Clinical presentation & parameters) and 3.4 (Co-morbid conditions and clinical management) are highly relevant as they describe the overall clinico-epidemiological profile of the entire hospitalized cohort (N=89). This context is essential for an NTD paper and sets the stage for the serotype-specific analysis (N=23).

Sample Size for Correlation (N=23): We agree completely. With DENV-1 and DENV-3 detected in only one patient each (4.3%), statistically robust associations cannot be drawn. We have revised the text in Section 3.5 (Dengue Virus Serotype & Clinical Features Distribution) and the Discussion to use cautious, descriptive language such as "observed symptoms in the limited non-DENV-2 cases" or "clinical profile noted during DENV-2 predominance," instead of language that implies statistical association.

6. I would not make any conclusions about molecular epidemiology with only 1 sequenced sample, especially since the authors were able to obtain high-quality data with only 1 of 23 samples with RT-PCR.

**Response:** This is a critical, justified critique. We acknowledge that one sequence is insufficient for drawing definitive conclusions about *molecular evolution* or *epidemiology*.

**Actions Taken:** The authors propose that the title can be changed from "molecular evolution" to "molecular characterization" to reflect the actual scope of the sequencing data. Also, the running title was modified accordingly. We have significantly softened the language in Section 3.6 (now *Phylogenetic Characterization*) and the *Discussion*. We now state that the single sequence provides a preliminary molecular snapshot which confirms the circulating genotype/lineage as DENV-2 and offers a potential indication of its regional relatedness to an isolated Bhopal strain. We have removed all language suggesting robust evolutionary conclusions in results and discussion sections. We explain that of the 23 RT-PCR positive samples, only one yielded high-quality sequence data. This low yield is attributed to the low concentration and fragmentation of viral RNA due to the late presentation of patients, a common challenge for molecular work in resource-limited settings. This is now fully explained in the *Methods* and *Limitations*.

10. The discussion includes multiple speculative statements that are not supported by the results.
**Response:** We have carefully revised the entire *Discussion* section, removing or tempering all speculative statements. Conclusions are now strictly restricted to the evidence presented on the predominant serotype (DENV-2), the clinical profile of the N=89 cohort, and the high proportion of late-phase diagnoses.

**Response to Reviewer 2 comments:**

1. Why were only 28 NS1-positive cases tested by RT-PCR? Could testing additional RNA-extractable samples have given a more complete picture of circulating serotypes?

2. Out of 794 dengue-positive cases, only 28 NS1-positive samples were tested by RT-PCR. Are these few samples really representative of all the cases? Would testing more NS1-positive (or possibly IgM-positive) samples have given stronger and more reliable conclusions about the circulating serotypes?

**Response 1 & 2:** We clarify that the study focuses on the hospitalized subset (N=89) of the total cases (N=794). The selection of only NS1-positive cases (N=28) for RT-PCR was a pragmatic decision due to limited residual sample volume/RNA quality/ load. Furthermore, NS1 positivity is the best proxy for peak viremia, increasing the likelihood of successful molecular amplification for serotyping. We frame our findings as a sentinel surveillance report providing the first molecular characterization from the region, not a statistically representative cross-section of all 794 cases, which is now explicitly stated as a limitation.

3. Sequencing Strategy: Only a subset of RT-PCR positive samples underwent sequencing. Please provide details on how samples were chosen for sequencing and whether this may have biased phylogenetic interpretation.

**Response:** Of the 23 RT-PCR positive samples, high-quality sequence data was only obtained from one sample (DENV-2). This was due to the low viral load/RNA integrity in the remaining samples, consistent with late-stage presentation. The selection was based on RNA concentration/quality required for Sanger sequencing, not on a prior clinical or demographic bias. We have acknowledged that this single sequence provides only a preliminary characterization, as discussed in response to Editor Comment 11.

4. Can the findings from this single tertiary care center be generalized to the wider population of northern Karnataka?”

**Response:** Authors agree that the findings cannot be directly generalized to the entire North Karnataka population. However, given that this tertiary care center serves as a major regional referral facility, our data provides a critical, first-ever local snapshot of the circulating serotype (DENV-2), which is valuable for public health surveillance in the region. The language has been adjusted in the Discussion.

4. Clinical Correlations: The manuscript reports symptom associations with serotypes (e.g., chills, retro-orbital pain with DENV-2). Given the small number of non–DENV-2 cases, how reliable are these associations?

**Response:** We agree that the number of non-DENV-2 cases (DENV-1 and DENV-3, N=1 each) is too small for statistical correlation. We have revised all text in Section 3.5 and the Discussion to avoid using terms like 'associated with' or 'suggesting,' instead focusing on a purely descriptive reporting of the observed clinical profile during the DENV-2 predominant season.

5. What specific factors determined which NS1-positive samples were chosen for further analysis?

**Response:** The NS1-positive samples represent the acute viraemia phase, which offers the highest probability of successful DENV RNA amplification and serotype determination.

6. NS1 antigen is typically detectable only during the first 5 days of illness. After this period, NS1 results may turn negative even in confirmed dengue cases. Do the authors have data on the number of days between symptom onset and sample collection? If available, please include this information to support the explanation for low NS1 positivity

**Response:** We do not have precise day-of-fever data for the entire cohort. However, the high IgM and low NS1 positivity strongly suggest presentation in the late viraemia phase (after day 5-7). This is a crucial finding and a limitation of our tertiary care setting. We have integrated this explanation into the *Discussion* to justify our diagnostic reliance on IgM and explain the low molecular positivity rate.

7. Hematological and Biochemical Parameters: Thrombocytopenia and creatinine levels are mentioned briefly but not fully discussed. Could the authors elaborate on the relevance of hematological and biochemical parameters, and indicate whether any kidney involvement was observed in severe cases?

**Response:** The hematological analysis revealed that thrombocytopenia an important feature in all serotypes. While Grade I thrombocytopenia (75,000–150,000/µL) was the most frequent finding among genotyped cases (10/23), both DEN2 and the single DEN3 case presented at the critical Grade IV level (<25,000/µL), indicating severe hematological compromise. The serotype 2 cases showed profound liver derangement, with SGOT levels reaching over 1100 U/L and SGPT over 640 U/L (S2 Table), indicating significant hepatic involvement. While generally stable, some patients, predominantly DENV-2, showed isolated acute kidney injury markers. (S2 Table).

8. For the samples that were tested for dengue serotypes, can the authors provide the corresponding CBC, RFT, and LFT results? Presenting this data in a table in the supplementary material would help readers see any clinical differences between serotypes.

**Response:** As suggested, a supporting information table (S1Table) has been created and included in the manuscript, summarizing the corresponding CBC, RFT, and LFT findings for the samples tested for dengue serotypes.

**Journal Requirements:**

1) Please ensure that the CRediT author contributions listed for every co-author are completed accurately and in full.
   At this stage, the following Authors/Authors require contributions: Rekha Patil, Asif Kavathekar, Madhav Prabhu, Madhavi Patil, Ameya Kejriwal, Kamran Zaman, Jyothi Bhat, and Subarna Roy. Please ensure that the full contributions of each author are acknowledged in the "Add/Edit/Remove Authors" section of our submission form.
   The list of CRediT author contributions may be found here: <https://journals.plos.org/plosntds/s/authorship#loc-author-contributions>

**Action taken:** All authors' CRediT contributions have been completed accurately and in full in the submission form.

2) Please provide an Author Summary. This should appear in your manuscript between the Abstract (if applicable) and the Introduction, and should be 150-200 words long. The aim should be to make your findings accessible to a wide audience that includes both scientists and non-scientists. Sample summaries can be found on our website under Submission Guidelines:
   <https://journals.plos.org/plosntds/s/submission-guidelines#loc-parts-of-a-submission>

**Action taken:** An author summary has been created and placed between the Abstract and the Introduction in the revised manuscript.

3) Please upload all main figures as separate Figure files in .tif or .eps format. For more information about how to convert and format your figure files, please see our guidelines: <https://journals.plos.org/plosntds/s/figures>

**Action taken:** All main figures have been uploaded as separate files in the required tif format

4) We have noticed that you cited Supplementary Figure 2 on page 8 in your manuscript. However, there is no corresponding file uploaded to the submission. Please upload it as a separate file with the item type 'Supporting Information'.

**Action taken:** The missing Supplementary Figure 2 has been modified as S2 Table and has been uploaded as a separate file with the item type 'Supporting Information'.

5) We have noticed that you have uploaded Supporting Information files, but you have not included a list of legends. Please add a full list of legends for your Supporting Information files after the references list.

**Action taken:** A full list of legends for the Supporting Information files has been added after the references list in the revised manuscript.

6) When completing the data availability statement of the submission form, you indicated that you will make your data available on acceptance. We strongly recommend all authors decide on a data sharing plan before acceptance, as the process can be lengthy and hold up publication timelines. Please note that, though access restrictions are acceptable now, your entire data will need to be made freely accessible if your manuscript is accepted for publication. This policy applies to all data except where public deposition would breach compliance with the protocol approved by your research ethics board. If you are unable to adhere to our open data policy, please kindly revise your statement to explain your reasoning and we will seek the editor's input on an exemption. Please be assured that, once you have provided your new statement, the assessment of your exemption will not hold up the peer review process.

**Action taken:** The statement has been revised to commit to making the data freely available upon request.

7) Please amend your detailed Financial Disclosure statement. This is published with the article. It must therefore be completed in full sentences and contain the exact wording you wish to be published.
   1) State the initials, alongside each funding source, of each author to receive each grant. For example: "This work was supported by the National Institutes of Health (####### to AM; ###### to CJ) and the National Science Foundation (###### to AM)."
   2) State what role the funders took in the study. If the funders had no role in your study, please state: "The funders had no role in study design, data collection and analysis, decision to publish, or preparation of the manuscript."

**Action taken:** The statement has been modified

8) Please revise your current Competing Interest statement to the standard "The authors have declared that no competing interests exist."

**Action taken:** The statement has been modified

9) Please ensure that the funders and grant numbers match between the Financial Disclosure field and the Funding Information tab in your submission form. Note that the funders must be provided in the same order in both places as well. Currently, "the Indian Council of Medical Research (ICMR)" is missing from the Funding Information tab.

**Action taken:** The statement has been modified

We are confident that these substantial revisions have addressed all the scientific and technical concerns and look forward to hearing from you soon.

Sincerely,

On behalf of all authors,

**Dr. Jyothi Bhat**

**Corresponding Author**
